# On Animal Excitability

**Published:** 1821-06

**Authors:** 


### Original Communications, Select Observations, etc.

---

### *On Animal Excitability.* By Dr. Kinglake.

ANIMAL excitability is a property inseparably connected with the living arrangement of matter. The innate powers of material agency are various, and are exerted according to the peculiar circumstances of organization in which they may be found to exist. It would seem that matter, in its minutest divisions, in its atomical as well as in its aggregate form, possesses an inherent motive power exerted in a repelling form, by which elementary and constituent substances are alike kept at certain distances, in the spheres or spaces of which the variously contrived agencies of life are incessantly performed. Whether this motive power be furnished by any generative faculty in the atomical and aggregated surfaces themselves, or be supplied by an animating principle or spirit with which all matter is, as it were, impregnated and vivified, and which becomes infinitely embodied and diversified agreeably to the particular arrangement into which it may enter, is more a question of theoretical refinement than of practical utility to determine. It is evident that the phenomena of organic life in existing circumstances cannot have a being without either the generative or mechanical influence of matter. To know the properties of life, therefore, in material arrangement, is an attainment of the highest importance; and to understand them in the various efficiency with which they are exerted, is to comprehend and distinguish the general and particular forms of animal existence. It is most clear that matter, whether originating or exhibiting life, is the only tangible and cognizable subject of reference, in contemplating and estimating the properties and conditions of the principle of animation.

During the continuance of life, the power by which the animal frame is impressed and affected by agents is appropriately termed excitability, because it refers to a condition that may be excited by the different agencies that are necessary to the living state. It is with this excitability that physiological speculations on the nature and properties of life are chiefly concerned. The general nature of this excitable property is shown by its diffusion over the whole animal system, enabling every part to be acted on agreeably to the scheme of life with which it is connected. This extended character of the property may be regarded as the constitutional excitability: it belongs to every part of the frame, and forms an aggregate of impressible or excitable power on which the nervous functions of life depend. Whether this excitability be the creature or the embodied cause of animal being, it matters not, with regard to its vital effervency: it is the great property with which medical inquiries have to do, and by which the validity of theories on the nature and cure of disease must be decided. The manner in which the excitability of organic life is distributed throughout every part of the frame, amidst the vast variety of structure and function that presents, evinces that its motive power is not limited to any part, but that it holds an undivided and an indivisible connexion with the whole. By this provision is secured an integrity in the phenomena of life, on which depend the order and harmony that characterize the healthful state of animal existence.

Animal life consists of a variety of functions; its objects are too numerous and diversified to admit of fewer offices. To furnish the means of executing what is necessary to life, parts are so constructed as peculiarly to fit them for the particular function they were respectively designed to perform. It is in this peculiarity of structure is found an excitability that may be denominated specific, because it possesses certain powers that enable it correctly to fulfil the intention for which it is provided. The dissimilar parts of the frame are all marked by conditions of excitability that adapt them to their destined uses. The structure that evolves an appropriate excitability, is an object deserving close attention, as being likely to unfold important truths, and to exhibit latent conditions of both the healthy and morbid states of life that indispensably require to be correctly known and duly regarded. The different textures of the animal frame are necessarily associated with such corresponding states of excitability as are requisite for the performance of their respective functions. The brain has its peculiar arrangement, its specific organization; and, in connexion with that structure, exists an appropriate excitability that constitutes the vital conditions and special motive powers of that organ. Its function is to feel, and to extend that power over the whole frame. On this sentient property, emanating from peculiarity of structure either generatively or mechanically, are founded the rational capabilities of the mind, and all the active energies of the body arising from the sensorial powers of animal life. An unequivocal, or even an intelligible, comprehension of the nature of that peculiar power by which the nervous structure performs its complicated function, is probably an arcanum that will remain locked up in the cabinet of nature, as a curiosity not necessary to be known, as an extent of research to which the prying inquisitiveness of man should not be carried; or, in other words, it is an object for the detection of which the structure itself does not appear to have furnished adequate means.

In the peculiar excitability occurring in different arrangements of organic structure, that of the visceral parts is most evident, and is connected with purposes of the greatest consequence to animal existence. The heart stands pre-eminently high in the scale of general, as well as particular, excitability; it may be justly regarded, perhaps, as the *primum et ultimum mobile* of animal life. Its structure endues it with its power, by furnishing it with an excitable aptitude for its great and unremitting function. The slightest deviation, whether in the excitable state or in the manner in which it may be excited, is attended with serious ailment,—an ailment felt at the very source of life,—at the origin of arterial action,—at the fountain from whence is derived the active energy that is intended to pervade the whole system. If this viscus be unduly excited by disease, or if its excitability should be altered and distempered by change of structure, no health can be experienced whilst such grievance remains. In common with all other textures, it is liable to change of tone, to oppressive fullness, to inflammatory action, to augmented and diminished bulk, to osseous conversion of structure, and even to ulcerative decomposition. This viscus, like others, too, has its appropriate stimulants, which are requisite to its healthy function,—such as the oxygenous principle of the atmospheric air, and having its cavities duly distended with the circulating fluids. Where these are wanting, disease arises; and, if furnished in excess, deranged health is equally the result. The provision for health which is laid in the fabric and correspondent excitability of the organ itself, admits of slight changes from the natural state, unconnected with altered structure, without danger. These changes, however, might induce morbid susceptibilities for becoming unduly affected. The excitable power might be either too readily or not sufficiently acted on; either immoderate or torpid action might equally, though differently, disturb the healthful state. The function of the heart, in all its relations to the several offices of the other viscera, and in all the morbid deviations to which it is liable consistently with the recoverable state, are objects well deserving the earnest attention of philosophical pathology.

The pulmonary structure is peculiarly adapted to the function of respiration. Its provisions for the distinct transmission of circulating and aerial fluids, are exquisitely contrived to insure an exact performance of the vital offices with which they are severally connected. From this peculiarity of structure results a condition of excitability, that precisely fits it for the purposes meant to be served by it in the animal economy. The standard and variable states of the specific excitability evolved by this structure, furnish a wide scope for the healthy and morbid action of the respiratory organs. Into these peculiarities researches should be carried, in correctly estimating the nature and tendency of pulmonary diseases. The membranous, vascular, vesicular, glandular, and cellular textures, of which the general pulmonary structure is composed, should be distinct objects of exact inquiry. When so much depends on diversity of structure in the relative actions of health and disease, it cannot be too minutely examined, nor its vital and morbid conditions be too closely scrutinized. The secret processes of nature, and the recesses in which they are carried on, are legitimate objects of inquiry; and they are not so hidden and inaccessible, but the labour in endeavouring to explore them may be rewarded, if not with complete success, most certainly by the acquisition of important additional knowledge.

The peculiar fabric entering into the organization of the stomach, presents a visceral arrangement of very singular and vast powers in the animal economy. The function of what may be called the gastric system, is elaborate in its contrivance and most important in its effects. The vital machinery adapted for such an office must necessarily be endued with excitable powers appropriate to such use. The precise nature of these powers, and the laws by which they are regulated, deserve the most correct attention. The stomach, as an original or insulated viscus, is of the utmost moment to life and health; and its relative and sympathetic connexions with other vital organs, and indeed with the various structures composing the animal frame, are highly influential in both the salutary and morbid actions of life. The gastric juice furnished by a secreting action performed by the fabric of the stomach, possesses, in its natural state, a solvent power, that is indispensably necessary to the digestive process carried on in the stomachic cavity. The fibrous, membranous, and cellular textures of the stomach, have respective uses assigned them, on the concert and accuracy of which the integrity of the gastric function essentially depends. The influence exerted by the excitable conditions of the stomach, under the vast range of susceptibility that may be induced by occurring circumstances, is too extensive to be defined. It obtains, more or less perceptibly, on all occasions of disease, going the length of affecting its healthy excitability. This will be either transient or permanent, according to the extent of impression that may have been made, or to the degree of deviation that may have been induced from the natural state. Whether original or sympathetic, therefore, the affections of the stomach are of the most serious concern in pathological consideration.

The intestinal canal is destined for digestive and transmitting offices; its structure, therefore, is adapted to these objects. It consists of membranous, cellular, and fibrous textures, which, in their separate and conjoint powers, possess excitable properties suited to the purposes for which they are designed. The constituent arrangement of this fabric generates the vital conditions on which the peculiar excitability and function of the intestinal portion of the first passages depend. To know minutely the structure of this part is important, as it suggests the extreme caution that should be observed in estimating the true cause and appropriate mode of treating the diseases to which it is liable. Anatomical knowledge unfolds lines, dimensions, figures, and textures, which are the legitimate grounds on which rational speculation may be raised as to the real nature of the living principle, in its various healthy and morbid states. A just conception of power, whether atomical, organic, or chemical, cannot be formed without understanding the precise machinery through which, or rather by which, it becomes operative. Effects cannot be comprehended without tracing them back to their causes, and thus unravelling the chain of influence by which they have been produced and manifested.

The liver is a viscus performing an important function in the animal economy. Its bulk is extensive, and is provided with a variously-tubulated and parenchymatous structure, adapted to the peculiar uses for which it is intended. It receives a large portion of the venous blood of the whole frame, from which is extracted, by a secerning process, the bilious fluid, which appears to be at once necessary for digestive purposes in the first passages, and to remove from the sanguineous mass alkaline principles, that would injure if retained in the system. The bilious fluid is materially, but not formally, present in the blood; the liver, therefore, by virtue of a vital process, for which it is fitted by peculiar organization, arranges the biliary constituents, and presents them in the formal state of bile.

The circumstances connected with the formation of bile in respect of the bulk of the hepatic organ,—the quantity of venous blood passing through it,—its comparatively slow progress,—its occasional detention, inducing congestive fullness and disease,—the low degree of irritable and sentient power in the liver, are peculiarities contrived for indispensable uses. The fabric that has furnished the mechanical requisites for giving effect to vital processes that will remove materials from the circulating mass that would be injurious if retained, and to arrange them into a compound that is necessary to the digestive function, and that also serves to regulate the refluence of the venous blood to the heart, is endowed with a structural excitability that renders it capable of exactly performing its allotted office; and the deviations from that healthy state of natural excitability, are shown in diseases that must always refer to the precise conditions that may have induced the morbid change. The liver is distinguished from every other visceral organ, by the vicarious office which it executes of transmitting more venous blood than belongs to its own fabric. It has its own arterial and venous blood in common with other viscera, but, superadded to this, it receives and forwards to the heart the refluent or venous blood of the abdominal, or chylopoietic, viscera. This auxiliary function associates it intimately and extensively with the healthy and diseased states of other parts; and therefore, perhaps, subjects it to a greater range and variety of morbid affection than is liable to befal any other vital organ.

The kidneys, with their appendages, the ureters, urinary bladder, and urethra, present a system of contrivance for the removal of the superfluous water from the mass of circulating fluids, that at once challenges admiration and merits the closest physiological attention. The construction of the renal and urinary machinary, like that of all other parts of the animal frame, is simple, and exquisitely adapted to fulfilling the purpose for which it is designed. The hepatic structure secretes, or forms, by a vital process peculiarly its own, the bilious fluid: the renal texture, exerting, in like manner, its peculiar powers, separates an aqueous fluid more or less impregnated with mucilaginous and saline materials, of an excrementitious nature, and which require to be periodically removed from the system. What is the peculiar structure that performs this function? The anatomist replies by demonstrating and detailing all that the knife can reach, and other devices for exploring hidden recesses can trace; but this knowledge, no more than that of the respectively peculiar fabric of the liver, brain, lungs, or any other visceral organ, affords no satisfactory solution of the question, how, or why, such an effect should result from such a cause. The principles of causation may be rigidly scrutinized, and all their authorities strictly applied, yet not a step is advanced in the inquiry beyond the simple knowledge that certain vitalized machinery produces definite effects necessary to animal life. *Causa latet, res ipsa notissima.* Like the liver, the lungs, and all other secreting organs, the kidneys have secerning vessels, by the exertions of which are effectuated the specific arrangement that constitutes the peculiar nature of the secreted fluid. Why should the *tubuli uriniferi* form urine? and why should they not? are corrolative questions, only answerable by a solution of the respective difficulties of the subject. They are endued with life, and placed in relative circumstances of organic connexion, of temperature, of chemical and mechanical influence, that would render such an effect inevitable. In all vital processes, an elective, assimilating, and an organizing power, is exerted, that can neither be controled nor imitated by artificial contrivances. It is necessary that the superfluous water of the blood, and other excrementitious materials of which the urinary fluid is the vehicle, should be seasonably removed from the system, or hurtful and eventually-destructive redundancy must speedily ensue. If any portion of the constituent substances of the blood should escape through the kidneys, it evinces a diseased state of the healthy secretion that should have obviated such an occurrence. The serous part of the blood, and even the blood itself, occasionally pass the urinary organ; as do also the same fluids, in hepatic diseases, through the biliary ducts of the liver. All secreting offices are executed by an appropriate mechanism, so vitalized as to possess the peculiar excitability necessary for the specific purpose meant to be effected.

The spleen is an organ executing, no doubt, an important office in the animal economy. Its obvious use is not so striking as that of other organs, which are evidently performing secreting or other functions. The glandular arrangement for extracting and transmitting some peculiar fluid from the blood passing through it, does not exist. Unlike either the uriniferous or biliferous tubes, it possesses no visible means of either forming or conveying any fluid that may have been secreted from its circulating blood. What, then, is its function? It is not a superfluous organ; it does not furnish an instance of being a work of supererogation, seated almost in the very centre of the animal frame. It is probably an auxiliary organ to the several functions of the stomach and liver, supplying these viscera with the additional, and perhaps modified, blood that is necessary to furnish the requisite quantity and quality of gastric and biliary fluids for digestive purposes. Alimentary substances would most likely not prove sufficiently nutritious to sustain the wear and tear of vital action, if the solvent power possessed by gastric and biliary fluids, secreted by respectively peculiar vessels, were not exerted on them. An adequate supply, therefore, of these agents is indispensably necessary to the intended effect. The spleen, not unlike the vena porta of the liver, for evident bilious purposes, would appear to furnish the necessary quantity of sanguineous fluid to the stomach to insure a due secretion of gastric juice for decomposing the dietetic substances which are used for nourishment. The spleen has a bluish appearance, and its texture is different from that of any other part. This peculiarity fits it for its specific use. Were its office that of an hydraulic machine, merely for the transmission of a fluid, that purpose might be effected by simple tubular arrangement; but the vital action of the spleen most probably induces such changes in its transmitted fluid as are necessary to the ulterior gastric and biliary secretions with which it seems to be connected. The peculiarity of fabric and living power likewise subjects the spleen to specific aberrations from the healthy state, and, in strictness, entitles it to the pathological discrimination that is due to every instance of dissimilarity in animal structure.

The pancreas is an abdominal organ, destined to aid in the grand work of digestion. It furnishes a fluid of an aqueous bland quality, resembling saliva, which serves to dilute the bile, and in other respects to render it more suitable to the digestive purpose for which it is employed. This auxiliary organ, like the spleen, does not exist in vain; it performs an important function that could not be effected in any other mode. The peculiarity of structure, fitting it for its specific office, induces certain vital differences, relations, and correspondences, with the general and particular mechanism of animal life, that render it a just object of physiological and pathological notice, in estimating its diseases and the most appropriate method of treating them.

The testes, the vesiculæ seminales, the uterus, and the ovaria, are parts concerned in the generative processes of animal being. If structure can be so vitalized as to be rendered capable of the vast diversity of function that presents in the animal economy, from the evolution of thought to the arrangement of saliva; if all that is admirable in contrivance, perfect in design, and exquisite in effect, can result from difference of fabric involving peculiar vital affinities or elective powers; then it may be easily conceived that the entire formation of the animal machine, in its fetal origin and progressive enlargement to the adult state, may be in the power of the genital organs, or parts especially constructed for that creative purpose. Whether the animal fibre be considered abstractedly as a simple elongation of organic matter, or regarded in all the various and dissimilar arrangements, combinations, and contextures, into which it is fabricated and distributed in the animal frame, whatever be the substance, and however constituted, it is equally liable to the inherent conditions of vital organization, determinable by the particular use it may be designed to fulfil. The external differences of animal organs are striking, but those which refer to the interior and evanescent structure are not less real; and it is in an accurate analysis of them that correct and efficient information is obtained concerning the precise power, agency, and effect, which they may be respectively capable of producing.

Inherent power is incessantly operative, it directly goes to its object: it may be perverted or misunderstood by erroneous speculations of its energies and uses, but it will still prevail; and all the effects which are exhibited in both the moral and physical world, may be justly regarded, however unperceived and unknown in numerous instances, as the unremitting exertions of its established influence. Correctly to know and appropriately to direct this power, form the rudiments of all science and art, of all natural and acquired intelligence. The regions of knowledge are infinite, but they are not dark and impenetrable; they may be explored and demonstrated by the light of truth, by which alone can be unveiled the minute and latent realities of inherent power in all the various arrangements of the material universe. To attempt a correct analysis of any species of power, whether atomical, chemical, mechanical, vegetative, or animal, is to undertake an arduous task. An outline and all the main relations of any particular power may be given; yet the more minute bearings, the recondite conditions on which specific properties and influence depend, are not so directly perceived, and are always rather objects of speculative calculation than of mathematical demonstration.

The great land-marks of general science, in common with these referring more particularly to the animal economy, are as evident as meridian light, and they infallibly supply, in spite of all misapprehension and sophistry, the information that is indispensably necessary for rational existence. They form, indeed, the exhaustless sources of intelligence incessantly furnished to common-sense inquiries; they are the alphabet of knowledge,—the straight-forward course that is at once discerned and understood. But this vast magazine of knowledge is composed of various parts, all which are fit objects of distinct research. The examination that disconnects and rejoins these constituent portions of knowledge, is instructive by developing powers, relations, properties, agencies, and effects, that emanate from circumstances which the decomposing aid of strict analysis could alone reveal. All things which exist are legitimate objects of inquiry, and they should, for purposes of correct and useful intelligence, be investigated in every absolute and relative situation appertaining to them with unsparing scrutiny.

*Taunton; March* 25, 1821.

